# Electrical signals dataset from fixed-speed and variable-speed synchronous generators under healthy and faulty conditions

**DOI:** 10.1016/j.dib.2024.111018

**Published:** 2024-10-10

**Authors:** Rafael Noboro Tominaga, Luan Andrade Sousa, Rodolfo Varraschim Rocha, Renato Machado Monaro, Sérgio Luciano Ávila, Maurício Barbosa de Camargo Salles, Bruno Souza Carmo

**Affiliations:** a University of São Paulo (USP), São Paulo, Brazil; b Federal University of Mato Grosso (UFMT), Cuiabá, Brazil; c Federal Institute of Santa Catarina (IFSC), Florianópolis, Brazil

**Keywords:** Power generators, Electrical currents, Signature analysis, Behaviour identification, Fault detection

## Abstract

Proper monitoring of rotating machines is responsible for the efficiency in detecting, diagnosing, or even prognosing failures. Effective monitoring can lead to increased economic viability of any equipment, as it may reduce costly repairs, decrease downtime, and increase safety. Knowing the behaviour of a machine promotes better monitoring of its operation and maintenance. Data-driven algorithms have been widely used to identify failures and predict the behaviour of machines and systems. The difficulty in obtaining reliable data to test strategies or methods for this purpose is well known. Our contribution is a set of electrical current data (time series data) from a rotating machine that generates electrical energy, generically called a power generator, in a laboratory. In this machine we have the possibility of, besides monitoring its healthy behaviour, causing internal defects that can reduce its efficiency and remaining useful life.

We highlight three key lines of study with the data available here: it is possible to apply data processing tools to make discoveries not evidenced in studies; test and compare new data-driven algorithms using public and reliable data; engineering lectures can use the dataset regarding the study of electrical machines and data driving methods.

The dataset contains information mainly about the voltage and current of generators when they are subject to internal faults. These faults include short circuits between turns of winding, short circuits between windings of the same phase, and short circuits between different phases.

This dataset has a wide variety of bench configurations. The dataset comes from real generators and allows the study of phenomena that are difficult to reproduce through analytical or computational models. The time series of electrical currents are raw, no preprocessing has been done. In fact, the signals contain natural noise from an industrial environment.

In this context, the main contribution of this work is to provide a public and reliable database, which helps to speed up the development of more efficient techniques for monitoring, diagnosis, and prognostics of the behaviour of rotating electrical machines.

Specifications Table

**Table:** 

Subject	Energy Engineering and Power Technology; Electrical and Electronic Engineering; Machine Design; Data Engineering; Signal Processing.
Specific subject area	Analysis of the electrical current signatures in rotating machines.
Type of data	Table, Figure Raw data, not analysed, not filtered, and not processed.
Data collection	The dataset was collected through benches with electro-electronic industrial sensors installed in specific positions to collect the variables of interest. The section's experimental design, materials and methods show the instrumentation used. The voltage sensors (LEM LV 20-P) have a measurement error of up to 2 % and the current sensors (LEM LTS 25-NP) 1 %. A data acquisition device can filter noise and assist in the file generation process. The tests were performed with at least one person present at the location, even with the development of a script to automate the execution of the rehearsals. The files were labelled according to the test conditions.
Data source location	Polytechnic School of the University of São Paulo, São Paulo, Brazil
Data accessibility	Repository name: Generators-Dataset Data identification number: 10.5281/zenodo.13685630 Direct URL to data: https://github.com/InnovaPower/MitDev-Eletrica Instructions for accessing these data: documentation in the same github.
Related research article	R. V. Rocha, L. A. Sousa, R. M. Monaro, Experimental Platform for Evaluation of Full-Scale Variable-Speed Wind Generators Under Stator Winding Faults, IEEE Transactions on Energy Conversion, 38, 2023, doi: 10.1109/TEC.2022.3230260.

## 1 Value of the Data

• Analytical models of variable speed synchronous generators under healthy and faulty conditions can be validated by comparison with laboratory data available here.
• It serves as a test and compares new data-driven algorithms using public and reliable data.
• Synthetic data can be generated in order to reproduce patterns and characteristics of the data available here. The idea is to be able to extrapolate the data to other operational conditions and nominal powers.
• This dataset can help solve overfitting problems in machine learning algorithms that aim to detect internal faults, because it can add more data to the training process.
• Engineering lectures can use the dataset regarding the effects of internal failures in generators and training machine learning algorithms, as well as validate mathematical models and simulations.

## 2 Background

These data were obtained from bench tests on generators connected to the electrical grid presented in studies conducted from Monaro (2013) to Rocha (2023), with some other researchers in between. Although each of the studies has a different method, since each authorʼs research proposal is different, they all have similarities in detecting faults through interpreting electrical features. Monaro (2013) uses fuzzy logic to improve the performance of real-time protection against faults of different topologies (phase-to-ground, between-turns, and between-paths) in electrical power systems. He ends up using synchronous generators in the two benches used for analysis. Rocha (2023) provides a similar analysis, but emphasising variable-speed synchronous generators connected to three-level converters. The difficulty in obtaining reliable data to test strategies or methods is well known. Our contribution is a set of electrical current data from a rotating machine that generates electrical energy. In this machine we have the possibility of, besides monitoring its healthy behaviour, causing internal defects that can reduce its efficiency and remaining useful life. In this context, we understand that the principal contribution is to provide a public and reliable database, which helps to accelerate the development of more efficient techniques for monitoring, diagnosis and prognosis of rotating electrical machines behaviours [[1], [2], [3], [4], [5]].

## 3 Data Description

All the important data descriptions are presented in this paper as a summary of original studies [1,2].

All tests have the common aim of investigating the behaviour of the system when the generator has internal faults, which are applied through the windings of the electrical machine. For this purpose, an experimental platform was developed: one with a synchronous generator with salient poles and fixed speed, another with a similar generator, but with smooth poles, and a third with a salient pole generator, but with variable-speed.

**Fig. 1:**
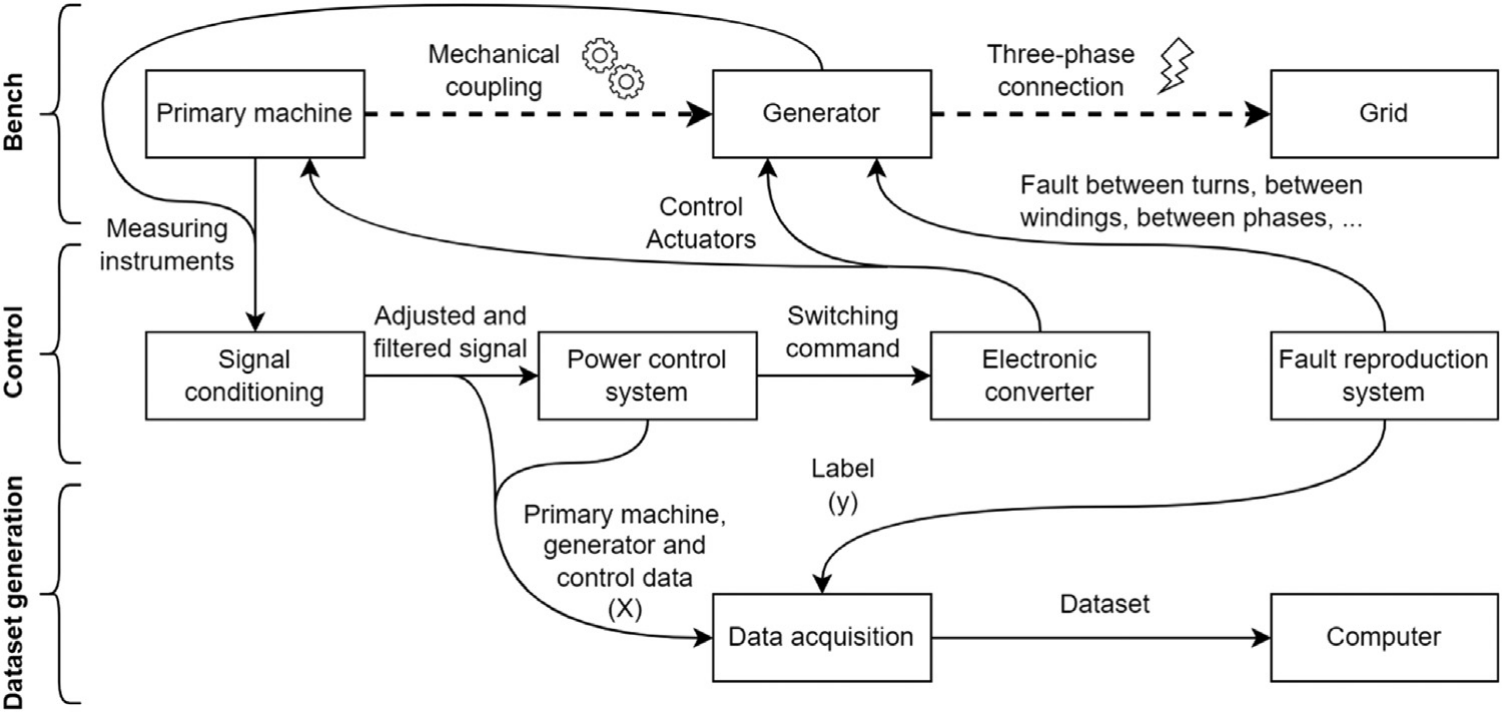
Block diagram of the experimental benches.

Fig. 1 presents a block diagram that shows the connections between each component used in the construction of the experimental benches. The illustration is reasonable for all benches presented in this study, which makes it possible to highlight the similarities. The power flow is represented by the dashed arrows, and these show that the energy is sent to the three-phase grid.

Among the components present on the bench, there is the primary machine, which aims to provide mechanical torque to the generator shaft. The primary machine can be a direct-current motor or even a three-phase induction motor controlled by a frequency inverter. The generator, connected to the primary machine through the mechanical coupling, converts mechanical energy to electrical energy. Finally, the electrical energy is treated before being sent to the grid to protect the grid and to approximate the real behaviour of a generator in the electrical power system with the experimental bench. This treatment ranges from the emulation of a transmission line through an equivalent impedance to the use of back-to-back converters to adapt the generation frequency to the grid frequency.

**Fig. 2:**
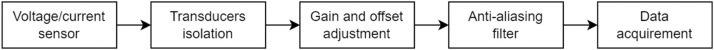
Block diagram of the sensor processing.

The bench cannot operate without the use of the control system. Note solid arrows represent the interaction between the control and the workbench, as well as the interaction of each component that makes up the control. The components that make up the control form a closed loop with the bench. The set of measuring instruments is mostly composed of voltage and current transducers. On some benches, an encoder is also used to measure the rotation speed of the generator shaft. Among the actuators are circuit breakers, contactors, and other devices responsible for controlling the benches. The signal conditioning system aims to isolate the entire control system from the power circuit, adjust, acquire and offset the collected signals and treat noise. All treated signals passed through a low-pass filter with a cutoff frequency of 2 kHz. Fig. 2 shows a block diagram of this processing. The power control system defines the values necessary for loop correction through switching commands. The electronic converter interprets these commands and, with the help of actuators, controls the active and reactive power of the bench.

**Fig. 3:**
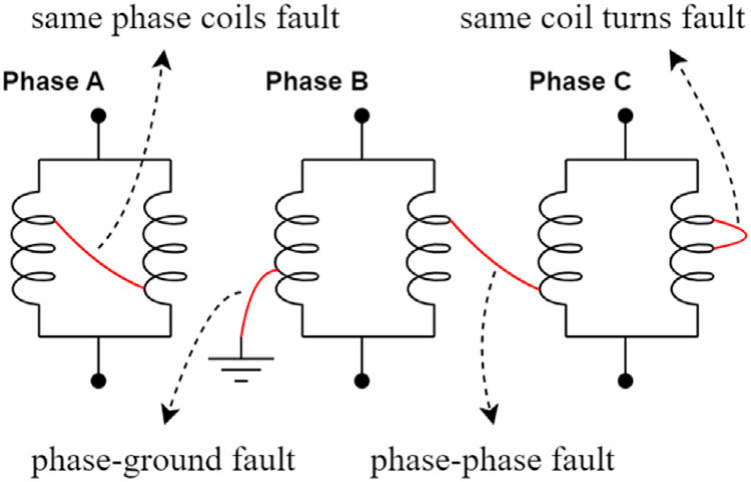
Faults explanation.

Both the control input and output information are used to generate the dataset. Data acquisition processes this information and sends it to the computer. The computer is used as an operator interface to operate the bench, where specific software is used for this process. The fault reproduction system aims to help change the generator windings and define the labels that each data must receive. The number of labels is defined by the number of faults that are inserted in the bench, among them, faults between turns, between windings, and between phases. Fig. 3 explains how to cause these faults and Table 1 shows which faults were caused for each bench. The “X” in the table shows the fault is done, and a dataset was obtained. It is important to highlight that the construction characteristics of some benches prevented all faults from being reproduced.

**Table 1:** Faults caused for each bench.

Bench	A	B	C
phase-ground	X	X	
phase-phase	X	X	X
same coil turns	X		X
same phase coils	X		X

Table 2 shows details of each block in Fig. 1 based on each bench. The first column indicates the block to be detailed and each of the other columns represents a different bench. There are three benches in total. In some lines, the cells are grouped, informing that all grouped benches share the same description.

**Table 2:** Specification of each block.

Bench	A	B	C
Local	Engineering School of São Carlos	Schulich School of Engineering	Polytechnic School of the University of São Paulo
Generator	Synchronous 4 salient poles	Synchronous 4 smooth poles	Synchronous 4 salient poles
Primary machine	DC motor with independent excitation		Three-phase squirrel cage induction motor
Mechanical coupling	Rigid		Belt and pulley systems
Three-phase connection	Equivalent impedance transmission line emulation, without converter		Two back-to-back converters of two (grid) and three (generator) levels
Measuring instruments	Measurement of voltage and current of the primary machine, the generator and the DC bus of the converters (when applicable) and angular speed of the generator shaft		
Signal conditioning	Isolation, gain and offset adjustment, anti-aliasing filter (2 kHz)		Gain and offset adjustment, low-pass filter (2 kHz) and buffer
Power control system	PI controller integrated into a own-designed dedicated control board (UCM)		PI controller made in MATLAB and Simulink and embedded in DSPACE
Data acquisition	PC architecture x86 with data acquisition		DSPACE Microlabbox

The link provided with the dataset shows four file folders. In three of them, they have the bench dataset, and the last one has the figures present in this paper. Table 3 shows general information about these folders. There are 2.62 GB and 4582 files.

**Table 3:** Dataset general information.

Folder name	Folder size	Number of files	Format
GEN_2KVA_4_SALIENT_POLES_FIXED_SPEED	146 MB	3314	csv
GEN_3KVA_4_SMOOTH_POLES_FIXED_SPEED	21 MB	637	csv
GEN_2KVA_4_SALIENT_POLES_VARIABLE_SPEED	2.45 GB	618	csv
Figures	10 MB	14	png

### 3.1 Benches A and B - Fixed speed generators dataset

**Fig. 4:**
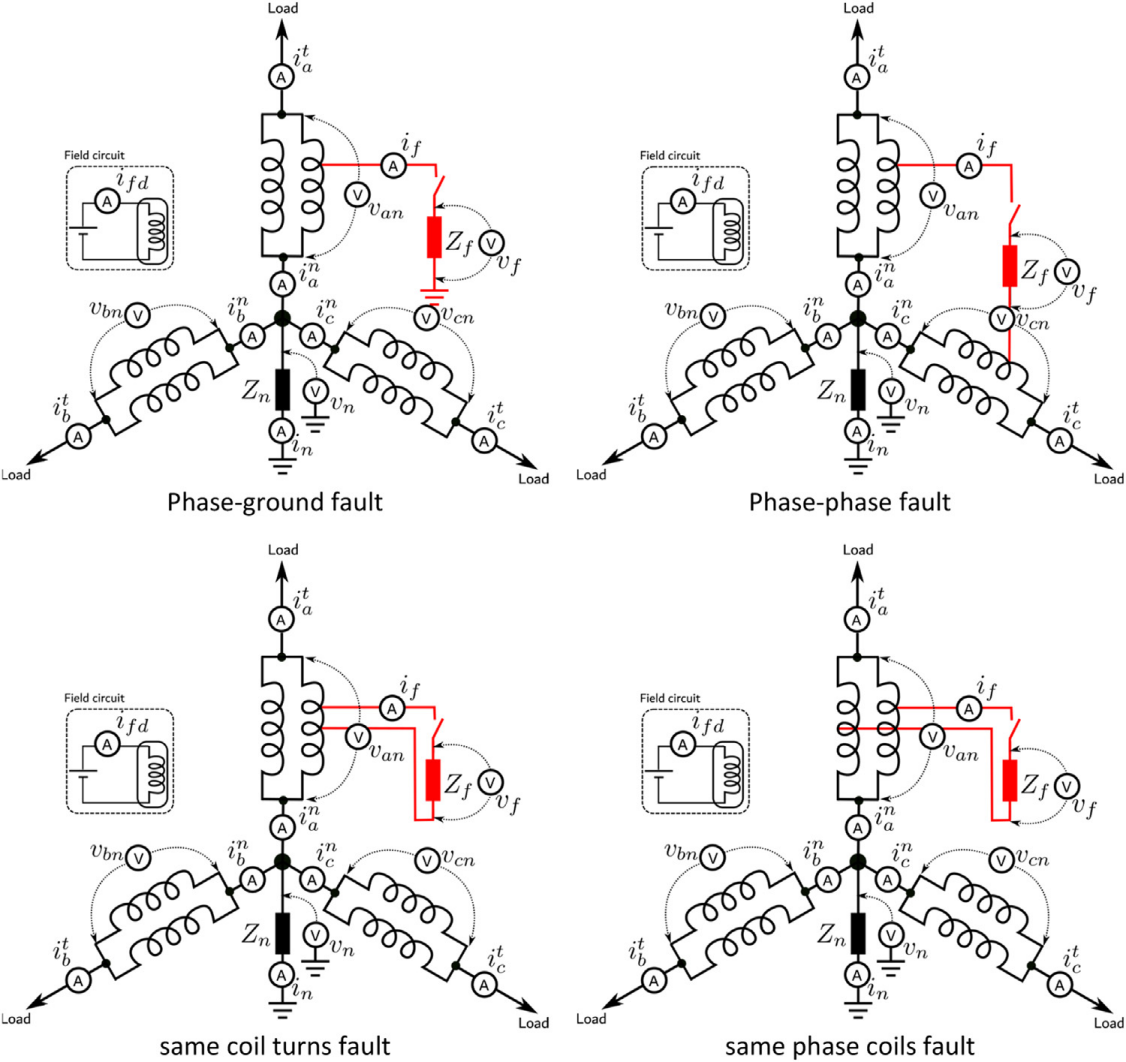
Variables measured for each fault condition.

Fig. 4 shows the positioning of the instrumentation responsible for generating the data set on the generator windings for each fault presented in Fig. 3. The windings of the three phases are presented 120° out of phase and connected in the grounded Y configuration. The voltage on each of the phases is measured, respectively, by $v_{an}$, $v_{bn}$ and $v_{cn}$. For each phase, two current measurements are taken. $i_{an}$, $i_{bn}$ and $i_{cn}$ between the windings and the neutral and $i_{at}$, $i_{bt}$ and $i_{ct}$ between the windings and their respective terminal. The purpose of this is to detect current leakage when the fault is executed.

For grounding, a neutral impedance of value $Z_{n}$ is used. The voltage applied to this impedance is measured by $v_{n}$, just as the current is measured by $i_{n}$. The fault is caused by the closure of the contact with impedance value $Z_{f}$. The voltage applied to this impedance is measured by $v_{f}$, just as the current is measured by $i_{f}$. Finally, the field circuit is presented to show that the $i_{fd}$ current is measured.

The list of features present in the data set is presented in Table 4. The first table column shows the position of the feature, the second the variable name based on the file header, the third the brief description and the last shows the variable unit. There is no missing value on the dataset. Approximately 260 ms were collected per data using a sampling time of 1024 µs. All data starts without the fault to avoid transient problems during acquisition (that is, fault switch boolean status is zero in time equal zero), and then the fault is entered only when the bench reaches steady conditions.

**Table 4:** Features description of fixed speed generator.

Column	Feature	Description	Unit
1	Time	Time elapsed after the start of recording.	s
2	VGERA	Phase voltage measured at phase A windings.	V
3	VGERB	Phase voltage measured at phase B windings.	V
4	VGERC	Phase voltage measured at phase C windings.	V
5	VN	Voltage measured at neutral and ground.	V
6	IGERAN	Phase A current measured at the neutral end.	A
7	IGERBN	Phase B current measured at the neutral end.	A
8	IGERCN	Phase C current measured at the neutral end.	A
9	IGERAT	Phase A current measured at the terminal end.	A
10	IGERBT	Phase B current measured at the terminal end.	A
11	IGERCT	Phase C current measured at the terminal end.	A
12	IN	Neutral to ground current measured.	A
13	IFD	Current measured at field circuit.	A
14	IFAULT	Current measured at fault impedance.	A
15	VFAULT	Voltage measured at fault impedance.	V
16	Speed	Rotor speed measured.	rad s^-1^
17	Active Power	Active power calculated according to voltage and current measurements.	W
18	Reactive Power	Reactive power calculated according to voltage and current measurements.	VAr
19	FAULT	Fault switch boolean status. 0 without fault. 1 fault.	–

The fixed speed smooth pole synchronous generator dataset files do not contain all the columns presented because, during the acquisition of this dataset, it was decided to keep the file sizes smaller; however, the columns present have the same meaning presented in Table 4. The missing features are the fault current and voltage, speed, and active and reactive power. Some of these columns can be recovered by doing mathematical operations using other columns.

### 3.2 Bench c - Variable-speed generators dataset

**Fig. 5:**
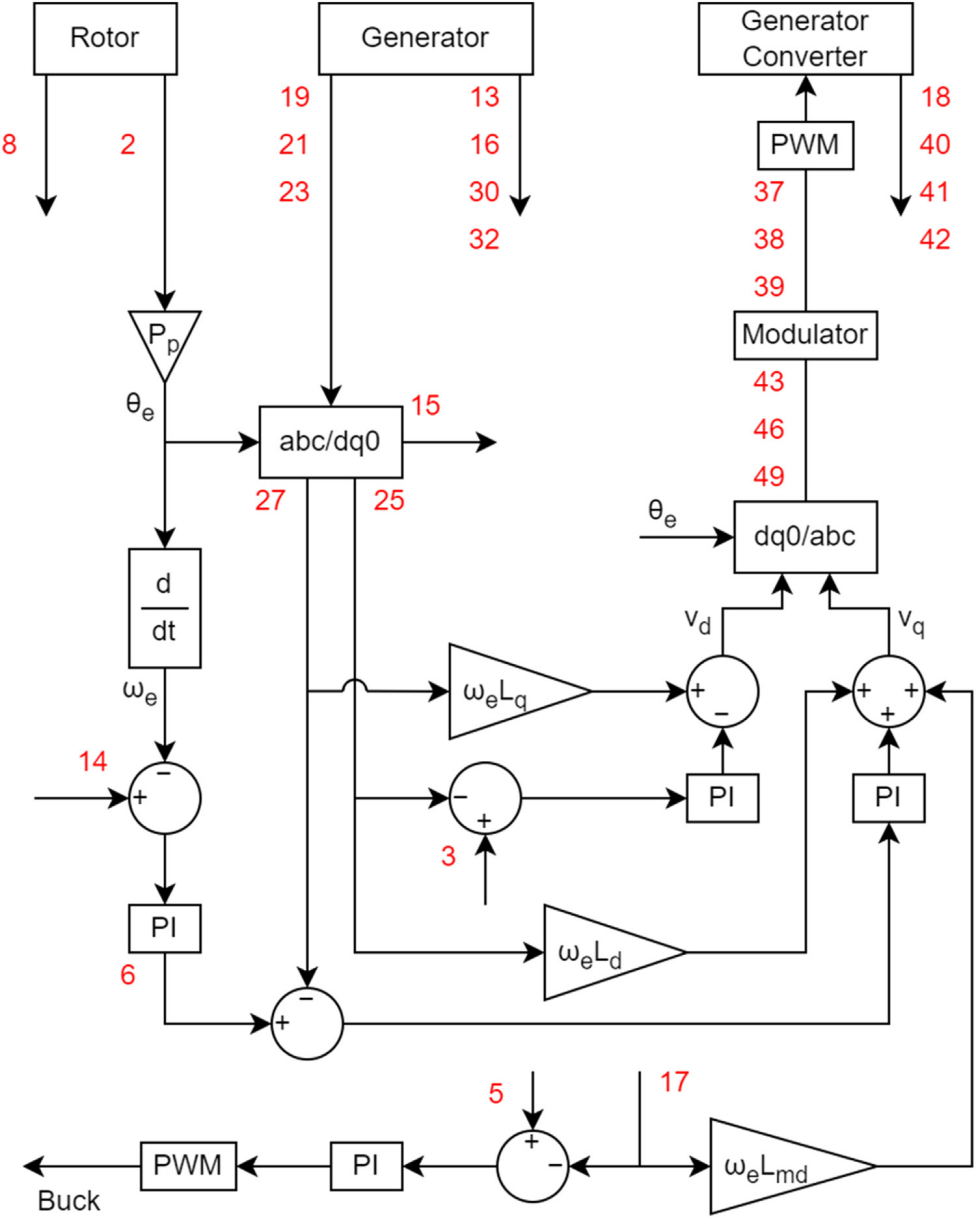
Generator control diagram and its measured variables.

**Fig. 6:**
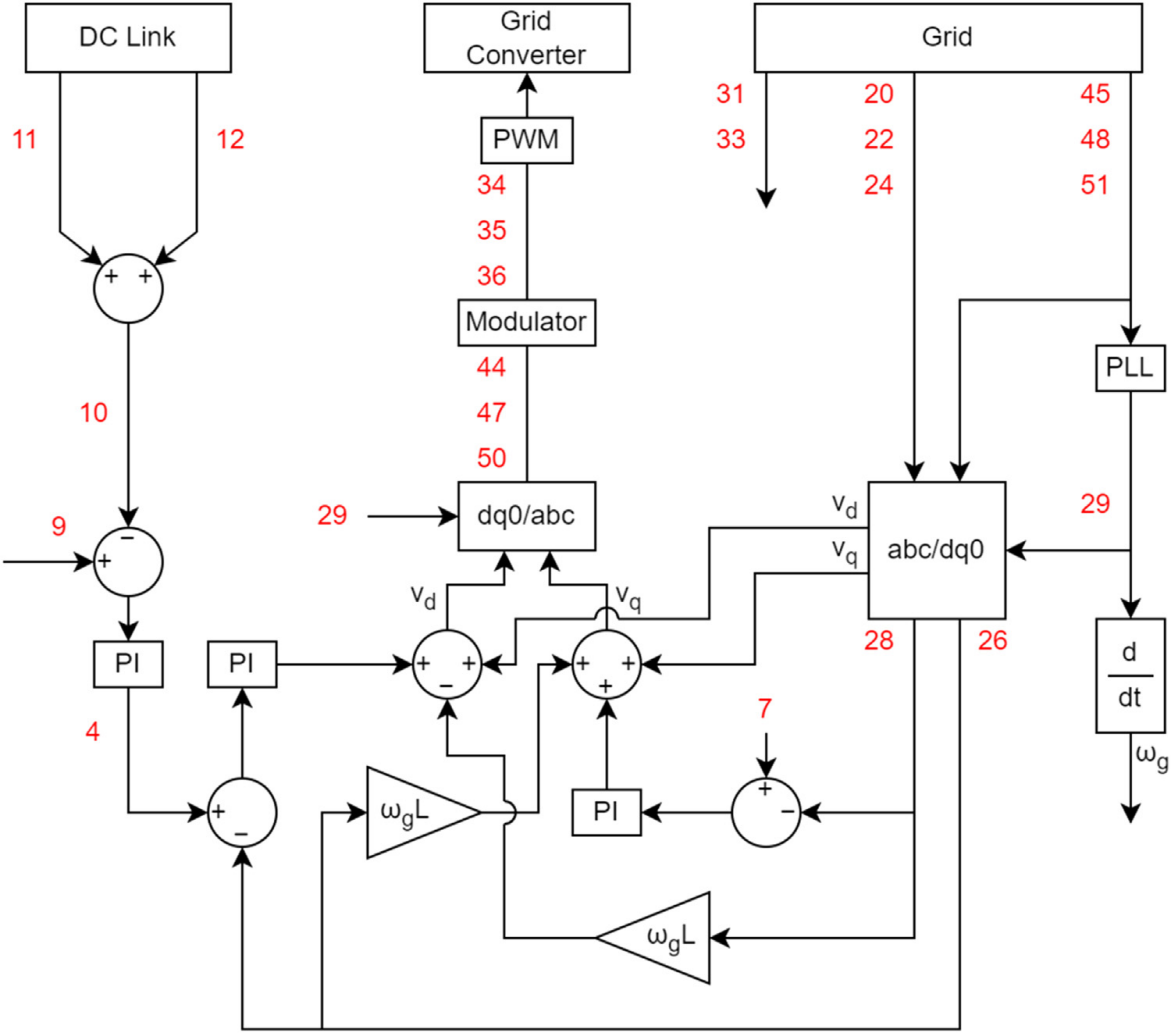
Grid control diagram and its measured variables.

Fig. 5, Fig. 6 do the same objective of presenting the variables as presented in Fig. 4. As there are many variables, in the diagrams they have been numbered to facilitate their identification. The diagrams also present the structure of the control used for the dataset acquisition. It is considered a Field-Oriented Control with rotor position measurement for the control of the variable-speed synchronous generator, and a Voltage-Oriented Control for the converter connected to the Grid. A Three-level Neutral-Point Clamped Converter was used for the generator control, so there are DC current and voltage measurements related to that type of converter.

The list of features present in the dataset is presented in Table 5. The meaning of the columns is like what was shown in Table 4. There is no missing value on the dataset.

**Table 5:** Features description of variable-speed generator.

Column	Feature	Description	Unit
1	Time	Time elapsed after the start of recording.	s
2	Ang_enc_cur	Angular phase difference measured by the encoder coupled to the rotor, wrapped between 0 and 2π.	rad
3	G_Id_gs_Ref	Direct-current reference for synchronous generator.	A
4	G_Id_grid_ref	Direct-current reference for grid.	A
5	G_If_ref	Current reference for field circuit of synchronous generator.	A
6	G_Iq_gs_Ref	Quadrature current reference for synchronous generator.	A
7	G_Iq_grid_ref	Quadrature current reference for grid.	A
8	G_Torque	Applied torque measured to the rotor.	N.m
9	G_VDC_Ref	Differential voltage reference for DC bus.	V
10	G_V_LinkDC	Differential voltage calculated at DC bus.	V
11	G_V_LinkDC_neg	Negative voltage measured at DC bus.	V
12	G_V_LinkDC_pos	Positive voltage measured at DC bus.	V
13	G_Vf_gen	Generator field voltage.	V
14	G_Spd_ref	Speed reference for generator.	rad s^-1^
15	I0_gen	Zero current calculed of the generator.	A
16	I_fault	Fault current measured.	A
17	If_gen	Field current of generator.	A
18	I_np	Three-level NPC Inverter neutral current measured.	A
19	Ia_gen	Current measured in phase A at the generator output terminals.	A
20	Ia_grid	Current measured in phase A at the grid output terminals.	A
21	Ib_gen	Current measured in phase B at the generator output terminals.	A
22	Ib_grid	Current measured in phase B at the grid output terminals.	A
23	Ic_gen	Current measured in phase C at the generator output terminals.	A
24	Ic_grid	Current measured in phase C at the grid output terminals.	A
25	Id_gen	Direct-current calculated of the generator according to current measurements.	A
26	Id_grid	Direct-current calculated of the grid according to current measurements.	A
27	Iq_gen	Quadrature current calculated of the generator according to current measurements.	A
28	Iq_grid	Quadrature current calculated of the grid according to current measurements.	A
29	Electric_Omega	Electrical angular phase difference used to calculate the dq0 transform.	rad
30	P_gen	Generator active power calculated according to voltage and current measurements.	W
31	P_grid	Grid active power calculated according to voltage and current measurements.	W
32	Q_gen	Generator reactive power calculated according to voltage and current measurements.	VAr
33	Q_grid	Grid reactive power calculated according to voltage and current measurements.	VAr
34	ma_grid	Phase A voltage modulated in the dq0 transform of the grid.	–
35	mb_grid	Phase B voltage modulated in the dq0 transform of the grid.	–
36	mc_grid	Phase C voltage modulated in the dq0 transform of the grid.	–
37	ma_gen	Phase A voltage modulated in the dq0 transform of the generator.	–
38	mb_gen	Phase B voltage modulated in the dq0 transform of the generator.	–
39	mc_gen	Phase C voltage modulated in the dq0 transform of the generator.	–
40	Vd_gen	Direct component of the generator voltage control.	V
41	Vq_gen	Quadrature component of the generator voltage control.	V
42	V0_gen	Zero component of the generator voltage control.	V
43	Va_conv_gen	Voltage measured in phase A at the point between the generator and the converter.	V
44	Va_conv_grid	Voltage measured in phase A at the point between the conversor and the grid.	V
45	Va_grid	Voltage measured in phase A at the grid terminal.	V
46	Vb_conv_gen	Voltage measured in phase B at the point between the generator and the converter.	V
47	Vb_conv_grid	Voltage measured in phase B at the point between the conversor and the grid.	V
48	Vb_grid	Voltage measured in phase B at the grid terminal.	V
49	Vc_conv_genr	Voltage measured in phase C at the point between the generator and the converter.	V
50	Vc_conv_grid	Voltage measured in phase C at the point between the conversor and the grid.	V
51	Vc_grid	Voltage measured in phase C at the grid terminal.	V

### 3.3 All benches - File description

**Fig. 7:**
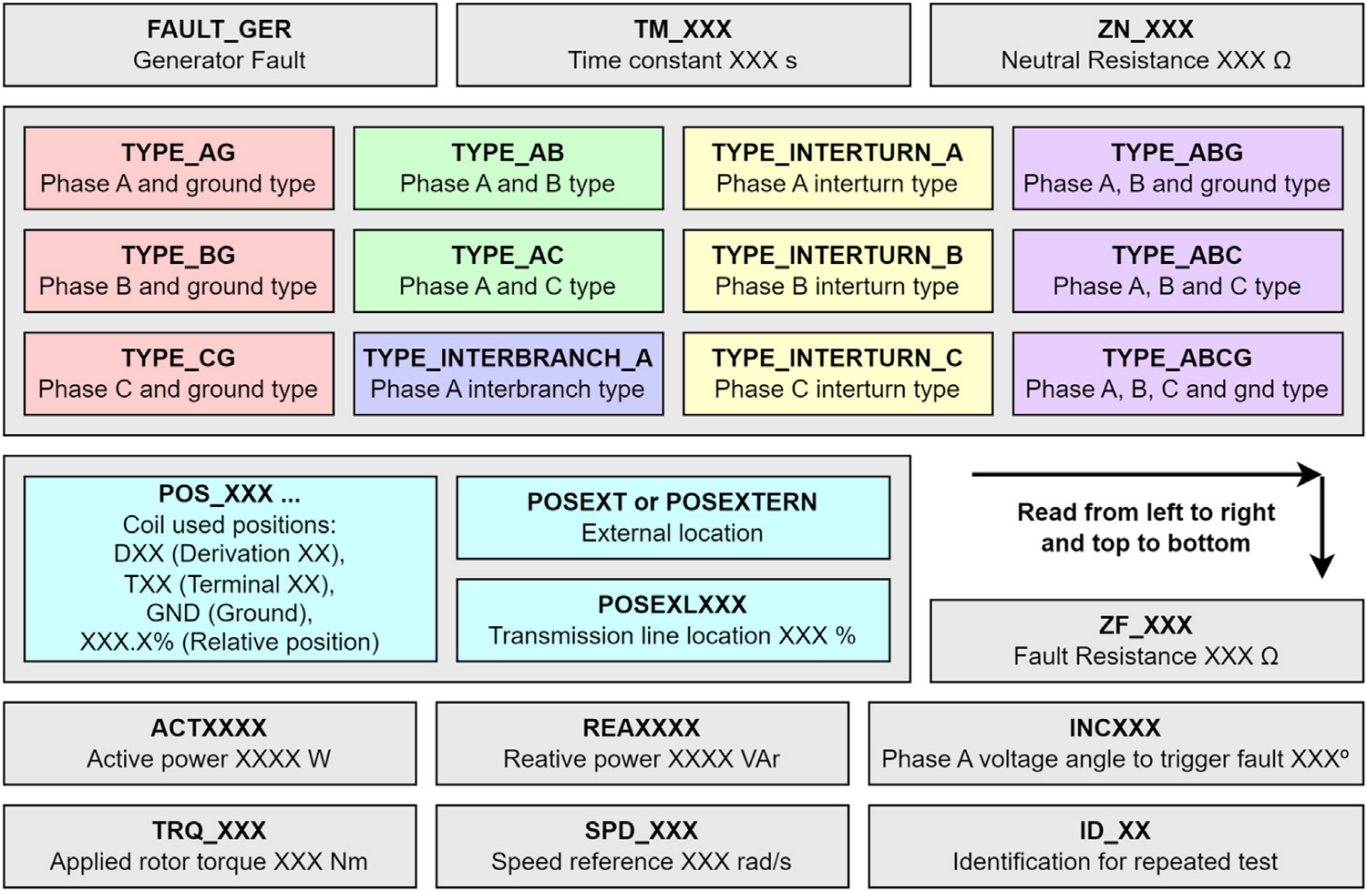
Files description of fixed speed generator.

The files were named in such a way that the test conditions could be extracted with no additional material. These conditions are represented through prefixes and suffixes linked to the type of fault. Fig. 7 shows precisely these prefixes and suffixes highlighted in bold and with a simplified description below. The grey rectangles represent a prefix or suffix that the file could contain, while the coloured rectangles represent the naming possibilities. The grey rectangles are organised according to the order of appearance of prefixes and suffixes in the filename, from left to right, and from top to bottom, as per the orientation shown in Fig. 7. Different colours were used for the faults to facilitate their classification according to the types presented in Fig. 3. It is worth mentioning that there is a group of faults not mentioned so far, which would be two-phase faults with ground and three-phase faults, with and without ground. These faults can be considered as a more advanced stage of the other faults already mentioned. The expression “XXX” represents a number.

**Fig. 8:**
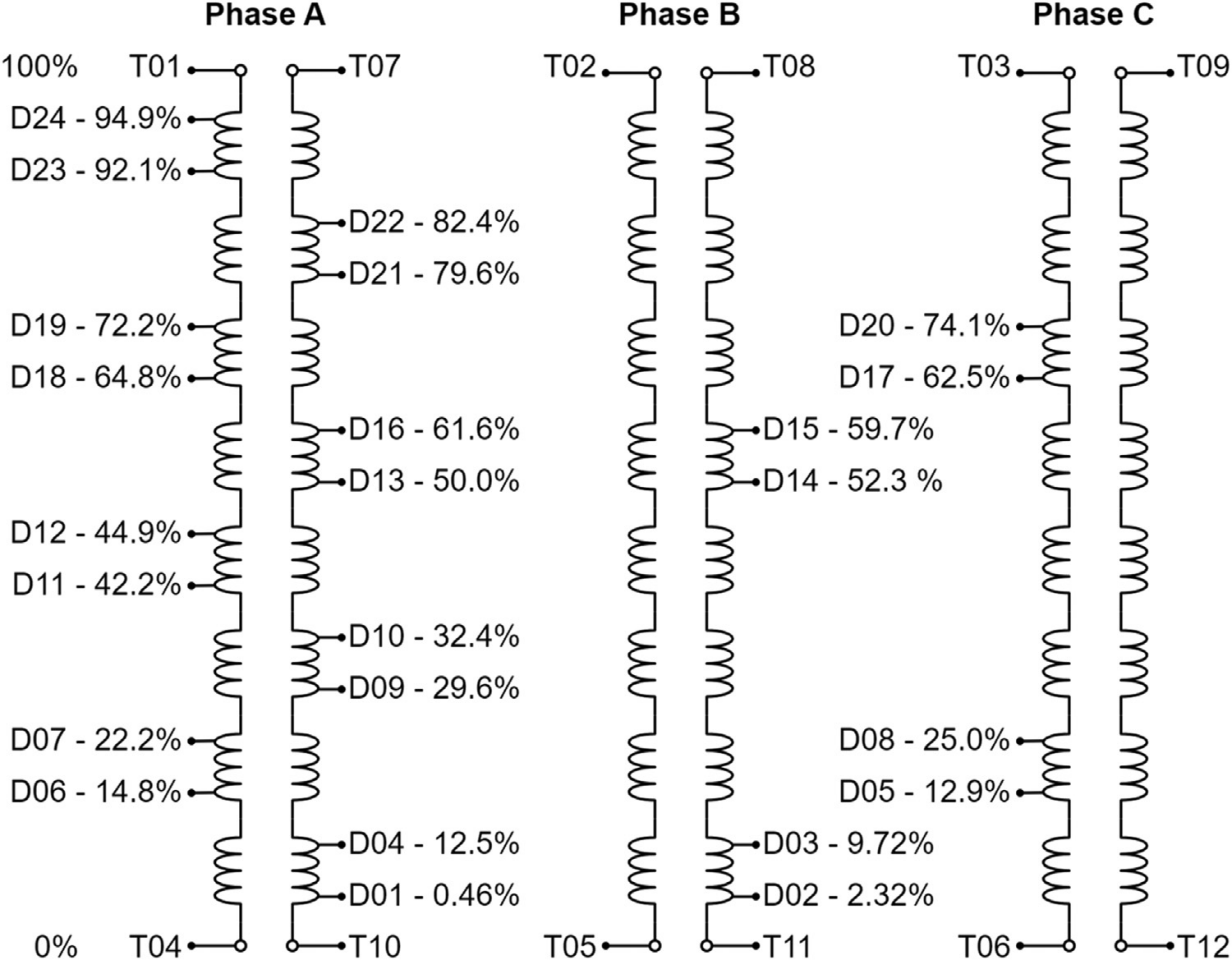
Explanation of the DXX and TXX of the windings.

Fig. 8 shows the fault insertion points for each of the windings. These points are represented by the suffix “POS_XXX” in Fig. 7. Each phase has two windings, and each winding has two terminals, which are represented by points T01 to T12. The windings have fault insertion points along their turns, which are represented by the other points D01 to D24. The percentage value that accompanies the points is related to the number of turns that are selected for the fault insertion.

## 4 Experimental Design, Materials and Methods

Once an overview of the benches has been presented, the following sections present more specific details of each of the benches. This is necessary because it is not possible to present all relevant information when aiming to generalise the structure of the benches as was done here.

### 4.1 Bench A - Salient pole synchronous generator with fixed speed

**Fig. 9:**
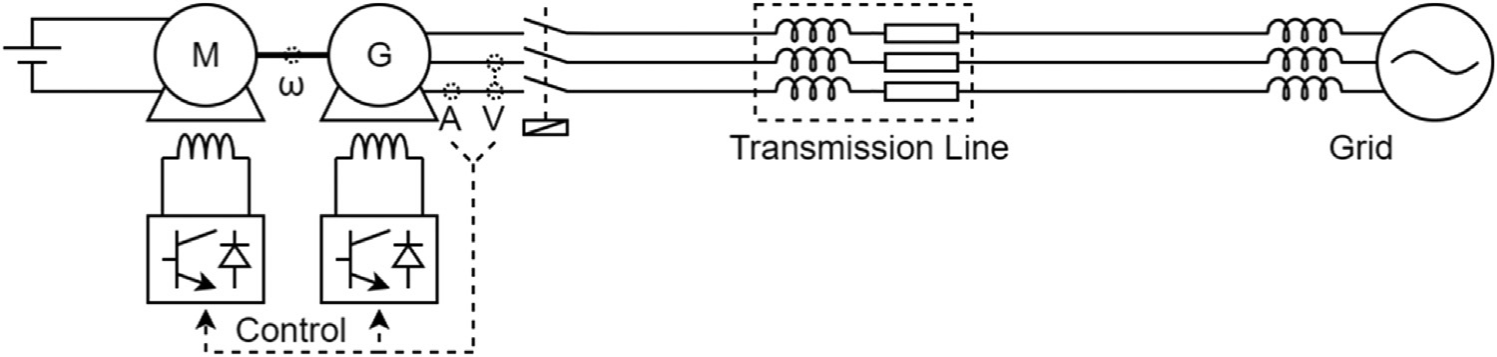
Electric diagram for salient pole synchronous generator with fixed speed.

**Fig. 10:**
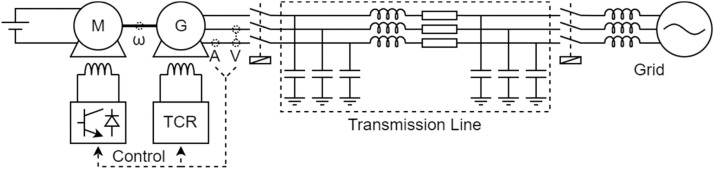
Electric diagram for Fixed speed smooth pole synchronous generator.

**Fig. 11:**
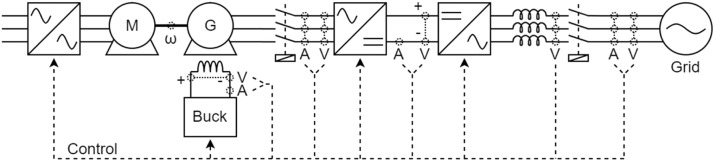
Electric diagram for Salient pole synchronous generator with variable-speed.

Fig. 9 shows the electrical diagram of the bench that contains the salient pole synchronous generator with fixed speed. To facilitate understanding of the diagram, the description of each component is performed following the power flow presenting the characteristics of the primary machine and ending by describing the network. Fig. 10, Fig. 11 also follow this behaviour during the description of the other electrical diagrams. These diagrams can be directly compared with Fig. 1.

The direct-current motor of power 1.7 kW, nominal speed of 1800 rpm, armature voltage of 250 Vdc, maximum armature current of 9 A, and maximum field current of 600 mA is used as a driving source for the generator. The motor was connected in an independent excitation configuration, in which its armature was powered by a 220 V direct-current source. Additional information is presented in Table 6 field voltage ($V_{fd}$), field resistance ($R_{fd}$), field inductance ($L_{fd}$), armature resistance ($R_{a}$), armature inductance ($L_{a}$), armature voltage ($V_{a}$), proportionality constant (K), moment of inertia (J), and viscous friction coefficient (B).

**Table 6:** Primary machine parameters of Bench A.

Parameter	Value	Parameter	Value
$V_{fd}$	170 V	$R_{fd}$	327.12 Ω
$L_{fd}$	81.78 H	$R_{a}$	1.95 Ω
$L_{a}$	33.75 mH	$V_{a}$	220 V
K	2.736	B	7.6E-3 kgm/rad s^-1^
J	0.0931 Nms²		

The synchronous generator has four salient poles, a terminal voltage of 220 V and a power of 2 kVA. Additional information is presented in Table 7 unsaturated direct-axis synchronous reactance ($X_{d}$), unsaturated direct-axis transient reactance ($X{{}^{\prime }}_{d}$), unsaturated direct-axis sub-transient reactance ($X^{\prime }{{}^{\prime }}_{d}$), unsaturated quadrature axis synchronous reactance ($X_{q}$), unsaturated quadrature axis sub-transient reactance ($X^{\prime }{{}^{\prime }}_{q}$), direct axis no-load time constant ($\tau_{d0}$), direct axis transient time constant ($\tau {{}^{\prime }}_{d}$), direct axis sub-transient time constant ($\tau^{\prime }{{}^{\prime }}_{d}$), zero sequence reactance ($X_{0}$), zero sequence resistance ($R_{0}$), negative sequence reactance ($X_{2}$), negative sequence resistance ($R_{2}$), field resistance ($R_{fd}$), armature resistance ($R_{a}$), and short circuit constant ($\tau_{a}$).

**Table 7:** Generator parameters of Bench A.

Parameter	Value	Parameter	Value	Parameter	Value
$X_{d}$	1.463 p.u.	$X{{}^{\prime }}_{d}$	0.163 p.u.	$X^{\prime }{{}^{\prime }}_{d}$	0.108 p.u.
$X_{q}$	0.778 p.u.	$X^{\prime }{{}^{\prime }}_{q}$	0.092 p.u.	$\tau_{d0}$	0.1669 s
$\tau {{}^{\prime }}_{d}$	0.0186 p.u.	$\tau^{\prime }{{}^{\prime }}_{d}$	0.0058 s	$X_{0}$	0.166 p.u.
$R_{0}$	0.106 p.u.	$X_{2}$	0.166 p.u.	$R_{2}$	0.106 p.u.
$R_{fd}$	242.26 Ω	$R_{a}$	1.5 Ω	$\tau_{a}$	0.00778 s

The generator output voltage and current are measured and used as input parameters for active and reactive power control. The active power is changed through the chopper in the field winding of the primary machine, while the reactive power is controlled through the chopper in the field winding of the generator. The choppers control the voltage that applies to the winding and have been integrated into a device dedicated to machine control.

As the bench does not have active elements capable of protecting the grid, an impedance of resistance of 0.81 Ω and inductance of 5.2 mH was added. This impedance aims to bring the test system closer to the real condition in which the synchronous generator is connected to the power system through a transmission line. The grid has an equivalent that is explicitly represented in the diagram because of the existence of the transmission line.

### 4.2 Bench B - Fixed speed smooth pole synchronous generator

Fig. 10 shows the electrical diagram of the bench that contains the fixed speed smooth pole synchronous generator. A direct-current motor with a power of 7.5 HP, nominal speed of 1800 rpm, voltage of 220 V, maximum armature current of 30 A, and maximum field current of 4 A was used as a driving source for the generator. The motor windings are configured in an independent excitation mode. The armature is fed by a 220 Vdc source and the field is through a 1-quadrant chopper that is connected to a 40 Vdc source.

The synchronous generator has four smooth poles, terminal voltage of 220 V, power of 3 kVA, and nominal current of 7.9 A. The armature winding of this generator has one path per phase composed of 4 coils in series. As it has a direct axis no-load transient time constant, that is much lower than the value found in large synchronous machines, a system called a time constant regulator increased this value. Additional information is presented in Table 8 unsaturated direct axis synchronous reactance ($X_{d}$), unsaturated direct axis transient reactance ($X{{}^{\prime }}_{d}$), unsaturated direct axis sub-transient reactance ($X^{\prime }{{}^{\prime }}_{d}$), unsaturated quadrature axis synchronous reactance ($X_{q}$), unsaturated leakage reactance ($X_{l}$), direct axis transient time constant ($\tau {{}^{\prime }}_{d}$), direct axis sub-transient time constant ($\tau^{\prime }{{}^{\prime }}_{d}$), and direct axis no-load transient time constant without time constant regulator ($\tau {{}^{\prime }}_{d0}$).

**Table 8:** Generator parameters of Bench B.

Parameter	Value	Parameter	Value	Parameter	Value
$X_{d}$	2.17 p.u.	$X{{}^{\prime }}_{d}$	0.375 p.u.	$X^{\prime }{{}^{\prime }}_{d}$	0.233 p.u.
$X_{q}$	2.17 p.u.	$X_{l}$	0.128 p.u.	$\tau {{}^{\prime }}_{d0}$	0.765 s
$\tau {{}^{\prime }}_{d}$	0.131 s	$\tau^{\prime }{{}^{\prime }}_{d}$	0.047 s		

Motor control was performed using the field weakening technique. A 1-quadrant chopper was used to control the voltage applied to the field winding. By controlling the field current, the torque exerted on the synchronous generator shaft can be adjusted, which therefore determines the active power injected by the generator into the network. A dedicated integrated circuit generates the PWM signal with a frequency of 5.0 kHz, which controls the static switches. This switching frequency suffices to guarantee continuous conduction of the generator field current. The duty cycle of the PWM signal was controlled by an input voltage of 10 Vp, coming from an analogue output of the data acquisition board. Control of the voltage applied to the synchronous generator field was done by the time constant regulator itself, whose input is directly connected to an analogue output on the data acquisition board.

A 500 kV, 100 MVA, double circuit transmission line with a length of 300 km was simulated by an equivalent composed of inductors, resistors and capacitors. The resistors and inductors were connected in series and represented the line cable, and the capacitors were connected between the cable and ground, representing the parasitic capacitance of the line. The values ​​of the line equivalent components were calculated so that, for a voltage of 208 V and power of 3 kVA, the line parameters are close, in p.u., to a real 500 kV line. The line equivalent was separated into 12 π sections, equivalent to 50 km each, composed of a resistor and an inductor in series and two capacitors connecting the end of each section to the ground. The grid also has an equivalent that is explicitly represented in the diagram because of the existence of the transmission line.

### 4.3 Bench C - Salient pole synchronous generator with variable-speed

Fig. 11 shows the electrical diagram of the bench that contains the salient pole synchronous generator with variable-speed. The three-phase induction motor with a “squirrel cage” rotor, nominal voltage of 220 V, and nominal power of 2 kVA is used as a driving source and is coupled to the synchronous generator shaft by a system of pulleys and belts. The motor is controlled by the inverter, which receives the reference value for torque control.

The generator has 4 salient poles, nominal power 2 kVA, nominal line voltage 220 V, and nominal field voltage 180 V. Additional information is presented in Table 9 base power ($S_{b}$), base voltage ($E_{b}$), base field current ($I_{fd}$), stator resistance ($R_{s}$), zero sequence resistance ($R_{0}$), negative sequence resistance ($R_{2}$), field resistance ($R_{f}$), unsaturated direct-axis synchronous reactance ($X_{d}$), unsaturated quadrature axis synchronous reactance ($X_{q}$), zero sequence reactance ($X_{0}$), negative sequence reactance ($X_{2}$), unsaturated direct axis transient reactance ($X{{}^{\prime }}_{d}$), unsaturated direct axis sub-transient reactance ($X^{\prime }{{}^{\prime }}_{d}$), unsaturated quadrature axis sub-transient reactance ($X^{\prime }{{}^{\prime }}_{q}$), proportionality constant ($K_{c}$), direct axis no-load time constant ($\tau_{d0}$), direct axis transient time constant ($\tau {{}^{\prime }}_{d}$), and direct axis sub-transient time constant ($\tau^{\prime }{{}^{\prime }}_{d}$).

**Table 9:** Generator parameters of Bench C.

Parameter	Value	Parameter	Value	Parameter	Value
$S_{b}$	2 kVA	$X_{d}$	1.463 p.u.	$K_{c}$	1.014
$E_{b}$	230 V	$X_{q}$	0.778 p.u.	$\tau_{s}$	0.00778 s
$I_{fd}$	209.1 mA	$X_{0}$	0.166 p.u.	$\tau_{d0}$	0.1669 s
$R_{s}$	0.056 p.u.	$X_{2}$	0.166 p.u.	$\tau {{}^{\prime }}_{d}$	0.0186 s
$R_{0}$	0.106 p.u.	$X{{}^{\prime }}_{d}$	0.163 p.u.	$\tau^{\prime }{{}^{\prime }}_{d}$	0.0058 s
$R_{2}$	0.106 p.u.	$X^{\prime }{{}^{\prime }}_{d}$	0.108 p.u.		
$R_{f}$	9.16 p.u.	$X^{\prime }{{}^{\prime }}_{q}$	0.092 p.u.		

The synchronous generator is connected to a Tree-Level Neutral Point Clamped inverter. The three-level converter connected to the generator has the following characteristics: nominal power of 10 kVA, maximum voltage of 600 Vdc, switching frequency of 10 kHz and IGBTs IRG4PF50WD. The fourth arm was used to supply the machine's field winding, and the others were connected to each phase of the stator. As the voltage supportable by the machine field is lower than the DC bus voltage, a DC/DC buck converter was implemented using the fourth arm switches.

The DC bus of this converter is connected to another two-level one. This second converter has the following characteristics: nominal power of 4500 VA, maximum voltage of 600 VDC, switching frequency of 10 kHz and IGBTs IRG4PF50WD.

The converter and machine control algorithms are embedded in hardware (Microlabbox) with the following technical characteristics: NXP (Freescale) QorlQ P5020 processor, dual-core, 2 GHz, 32 kB L1 data cache per core, 32 kB L1 instruction cache per core, 512 kB L2 cache per core, 2 MB L3 total cache for real-time operation, NXP (Freescale) QorlQ P1011 processor, 800 MHz for computer communication, Xilinx® Kintex −7 XC7K325T FPGA, 1GB DRAM and 128 MB flash memory, analog inputs and outputs with 16-bit resolution and ±10 *V* scaling and digital inputs and outputs with up to 5 V scaling and 10 ns resolution.

## Limitations

The dataset is limited by measurements from only three low-power machines. Additional datasets may be required to achieve satisfactory performance in the proposed applications presented in Value of the Data. It was not possible to perform tests and acquire data on high-power machines due to physical installation and safety limitations. The intensity and duration of faults were controlled so as not to compromise the integrity of the equipment.

## Ethics Statement

Our research adheres to the ethical requirements for publication in Data in Brief, does not involve human or animal subjects, and no data has been collected from social media platforms.

## CRediT authorship contribution statement

**Rafael Noboro Tominaga:** Data curation, Investigation, Writing – original draft. **Luan Andrade Sousa:** Data curation, Investigation. **Rodolfo Varraschim Rocha:** Data curation, Investigation, Writing – review & editing. **Renato Machado Monaro:** Conceptualization, Methodology, Data curation, Investigation, Writing – review & editing. **Sérgio Luciano Ávila:** Conceptualization, Writing – review & editing. **Maurício Barbosa de Camargo Salles:** Conceptualization, Project administration. **Bruno Souza Carmo:** Conceptualization, Project administration, Writing – review & editing.

## Data Availability

GithubGenerators-Dataset (Original data).
